# Correlation of breathing task derived cerebrovascular reactivity with baseline CBF, OEF and CMRO_2_

**DOI:** 10.3389/fneur.2025.1534844

**Published:** 2025-10-10

**Authors:** Ke Zhang, Simon M. F. Triphan, Mark O. Wielpütz, Johan Jende, Emilie Sleight, Christian Herbert Ziener, Mark E. Ladd, Heinz-Peter Schlemmer, Hans-Ulrich Kauczor, Oliver Sedlaczek, Felix T. Kurz

**Affiliations:** ^1^Department of Diagnostic and Interventional Radiology, Heidelberg University Hospital, Heidelberg, Germany; ^2^Department of Diagnostic Radiology and Neuroradiology, University Medicine Greifswald, Greifswald, Germany; ^3^Division of Radiology, German Cancer Research Center, Heidelberg, Germany; ^4^CIBM Center for Biomedical Imaging, Geneva, Switzerland; ^5^Division of Medical Physics in Radiology, German Cancer Research Center, Heidelberg, Germany; ^6^Division of Neuroradiology, Geneva University Hospitals, Geneva, Switzerland

**Keywords:** cerebrovascular reactivity, cerebral blood flow, oxygen extraction fraction, cerebral metabolic rate of oxygen, correlationship

## Abstract

**Rationale and objectives:**

Only a few studies examined the correlation between cerebrovascular reactivity (CVR) and other physiological parameters such as cerebral blood flow (CBF), oxygen extraction fraction (OEF) and cerebral metabolic rate of oxygen (CMRO_2_). In this study, these baseline parameters were measured using 3D MRI with whole brain coverage for the investigation of global and regional correlation between each other to enhance understanding of brain function and improve tumor diagnosis.

**Materials and methods:**

All measurement were performed at 3 T. CVR was derived from a breath-holding task. Baseline CBF was measured by pseudo-continuous arterial spin labeling. Baseline OEF was measured with a gradient-echo sampling of spin-echo pulse sequence. T1 weighted anatomical image (T1W) was measured using MPRAGE sequence. CVR was calculated using customized written programs. CBF was quantified by using ASLtbx. For OEF analysis, a feedforward artificial neural network was used. CMRO_2_ was calculated based on smoothed and normalized CBF and OEF. General linear regression analysis was used to examine the relations between CVR and other parameters in five lobes of gray matter including frontal, parietal, temporal, occipital and insula lobes in individual healthy subjects. Spearman correlation was performed to check the regional correlations in an Automated Anatomical Labeling (AAL) atlas.

**Results:**

Fifteen healthy volunteers and five patients with brain tumors were included. In the healthy subjects, five lobes had a positive correlation between CBF and CVR (*p* < 0.05). Similarly, in five lobes positive correlations between CMRO_2_ and CVR were found (*p* < 0.05), as well as significant inter- and intra-subject correlations (*p* < 0.001). However, there were no significant correlations between OEF and other parameters.

**Conclusion:**

Our findings demonstrate that CVR is strongly associated with CBF and CMRO₂ at both global and regional levels in healthy brains, but not with OEF. These results provide new insight into the complex interplay between vascular reactivity, perfusion, and metabolism and underscore the potential of combined CVR-CBF-CMRO_2_ imaging for assessing brain health and pathology.

## Introduction

Cerebrovascular reactivity (CVR) is an index of the brain vessels’ dilatory capacity, and is typically measured using hypercapnic gas inhalation or breath-holding as a vasoactive challenge (1–3). Research indicates that the baseline vascular state is associated with CVR. During task-induced and resting states, changes in CVR were shown to be modulated by the vascular and metabolic baseline states (4–9). The vascular and metabolic baseline states includes cerebral blood flow (CBF), oxygen extraction fraction (OEF) and cerebral metabolic rate of oxygen (CMRO_2_).

Measurements of these parameters have been performed in the analysis of brain metastases (10), calibrated fMRI (11), or for the effect of acetazolamide administration using ^15^O PET (12). However, a limited number of studies have investigated the correlation, particularly the regional correlation, of CVR with other physiological parameters such as OEF and CMRO_2_ (13, 14). Previous research (7) has shown a significant association between task-related fMRI responses and underlying physiological factors at rest, such as CVR and baseline venous blood oxygenation (*Y_v_*). Another study (6) explored similar physiological influences on resting-state fMRI metrics, revealing their significant impact on both resting-state functional activity (RSFA) and functional connectivity (FC) measurements. Additionally, other reports showed a positive correlation between baseline cerebral blood flow (CBF) and CVR, both between- and within-subjects (15–17). Whole brain physiological parameters such as global CBF, global venous oxygenation (*Y_v_*), RSFA, and CVR have been measured and studied (6, 7). Baseline *Y_v_* was determined in the superior sagittal sinus using the T2-Relaxation-Under-Spin-Tagging (TRUST) technique (8), CVR was measured using inhalation of 5% CO_2_ gas, RSFA was eventually obtained, and whole brain baseline cerebral blood flow (CBF) was measured using phase contrast (7). It was shown that the fMRI signal amplitude was positively correlated with CVR and RSFA, but negatively correlated with baseline *Y_v_*. Furthermore, among the physiological modulators themselves, significant correlations were observed between baseline *Y_v_* and baseline CBF, and between CVR and RSFA, suggesting that some of the modulators may partly be of similar physiological origins.

Regional correlations among these physiological parameters may help to elucidate the metabolic demands of different brain areas. Additionally, they may shed light on how these areas respond to changes in blood flow and oxygen supply. In this study, these baseline physiological parameters were measured using MRI covering the full brain. We calculated regional and global correlations between breathing task-derived CVR and baseline CBF, OEF, and CMRO_2_ in healthy volunteers and in patients with tumors. Our main objective was to investigate correlations among CVR, CBF, OEF, and CMRO_2_ in healthy individuals across lobes of gray matter, as well as to assess their variations between and within subjects. Additionally, we included a small cohort of brain tumor patients to evaluate the feasibility of the method in a clinical setting and to identify potential correlations among individual parameters within the tumor region, as previously described (18).

## Methods

Fifteen healthy volunteers (7 female, 8 male, aged 30 ± 5 years) were examined prospectively using a 20-channel head coil on a 3 T scanner (Magnetom Prisma, Siemens Healthineers, Erlangen, Germany). All participants provided written informed consent, and the study was approved by the institutional ethics committee. Additionally, five patients with brain tumors were measured using a 64-channel head coil on the same scanner.

To measure CVR, breath-hold (BH) respiratory challenges were integrated into a standard clinical brain imaging protocol: for block-designed BH tasks, 110 measurements were obtained, which include five and a half BH/FB (free breathing) cycles with 20 measurements (34 s) per full cycle and 10 measurements (17 s) per half cycle. The duration of breath hold time and free breathing time were both 17 s. To measure baseline CBF, a pseudo-continuous arterial spin labeling (pCASL) sequence with 3D gradient- and spin-echo imaging (GRASE) readout was applied before BH tasks. Presaturation before labeling and background suppression during a postlabeling delay (PLD) were added. Baseline OEF was determined based on a gradient-echo sampling of spin-echo (GESSE) pulse sequence (19). The baseline CMRO_2_ was then calculated from CBF and OEF. Specific sequence parameters and data analysis are as follows.

CVR (2D gradient-echo EPI): FOV = 220 × 220 × 118 mm^3^, matrix size = 64 × 64 × 28, resolution = 3.4 × 3.4 × 3.5 mm^3^, slice gap = 0.7 mm, in-plane iPAT factor = 2, multiband factor = 2, bandwidth = 1776 Hz/px, TE = 27.08 ms, TR = 1700 ms. Total acquisition time = 3.12 min. Preprocessing including motion correction and slice timing correction was performed with SPM12 (Wellcome Trust Centre for NeuroImaging, UK) and postprocessing was implemented using customized programs written in MATLAB (MathWorks, Natick, MA, USA). In conventional CVR analysis, measurements of end-tidal CO_2_ (Et-CO_2_) are required and used in a linear regression equation (20). Since we did not have Et-CO_2_ measurements available, CVR was estimated by replacing Et-CO_2_ with the mean signal of gray matter (21). Motion correction using SPM including realignment and reslicing and nuisance regression was performed to remove the motion corruption induced by the breath-holding task.

Baseline CBF (3D pCASL GRASE): FOV = 220 × 220 × 120 mm^3^ matrix size = 64 × 64 × 24, resolution = 3.4 × 3.4 × 5 mm^3^, slice and plane partial Fourier = 6/8, slice oversampling = 16.7%, FA = 120°, segments = 2, Bandwidth = 2,298 Hz/px, labeling duration = 1.8 s, PLD = 1.8 s, TE = 17.18 ms, TR = 4,500 ms. This sequence had 20 tag and 20 control volumes and one M_0_ volume, for a total scan time of approximately 7 min. CBF was calculated using ASLtbx (22). A labeling efficiency of 0.86 was assumed in the calculation.

Baseline OEF (GESSE): FOV = 256 × 192 × 117 mm^3^, partial Fourier = 6/8, matrix size = 128 × 96 × 30, resolution = 2 × 2 × 3 mm^3^, slice gap = 0.9 mm, TE = 51 ms; TR = 105 ms, number of total echoes = 64, number of echoes before echo center = 20, averages = 3, acquisition time was about 10 min. For OEF analysis, a feedforward ANN (artificial neural network) was used because of its high capability for nonlinear regression problems (23). Here, an ANN was chosen for its robust curve-fitting, good resilience to noise and outliers, and superior computational speed compared to conventional least-squares regression (LSR) methods. The ANN consisted of a 64-dimensional input layer, two hidden layers (32 and 10-dimensional), and a 4-dimensional output layer. Itwas implemented using the Neural Network Toolbox provided by MATLAB. The ANN was trained based on the full quantitative BOLD (qBOLD) model (24). Thereby, artificial GESSE signals with a known ground truth were simulated for plausible ranges of the input variables *S_SE_, R_2_, λ,* and OEF. To reduce overfitting artifacts, noise was added to the simulated GESSE signals before the actual ANN training (23) was initialized.

T1 weighted anatomical image (T1W): FOV = 230 × 230 × 176 mm3, matrix size = 320 × 320 × 176, resolution = 0.4 × 0.4 × 1 mm3, in-plane iPAT factor = 2, slice partial Fourier = 6/8, TE = 2.63 ms, TR = 1700 ms, TI = 900 ms, FA = 8°, Bandwidth = 2,298 Hz/px, acquisition time was about 5 min.

The CVR and CBF maps were smoothed using a Gaussian kernel with full width at half maximum of 4 mm. Next, all images were coregistered to the T1W and normalized to the Montreal Neurological Institute (MNI) standard brain space for healthy subjects.

Baseline CMRO_2_: After smoothing and normalization of CBF and OEF into MNI space, CMRO_2_ was calculated as (25):


$$\mathrm{CMR}O_{2}=\mathrm{CBF}.\mathrm{OEF}.{\left[H\right]}_{a}.$$

The oxygenated heme molar concentration in arterioles *[H]_a_* was assumed to be 7.377 μmol/mL (24).

### Statistical analyses

General linear regression analysis was used to examine the relations between CVR and other parameters in five lobes of gray matter including frontal, parietal, temporal, occipital and insula lobes in individual healthy subjects. Parameter maps were averaged across healthy subjects to compute group-level histograms in the whole brain, gray matter (GM) and white matter (WM). Spearman correlation was performed to check the regional correlations in an Automated Anatomical Labeling (AAL) atlas with 116 indexes. In order to extract the top 12 (i.e., 10% of 116) correlation regions, AASL regions with the lowest *p*-value were selected and plotted. For the patients, tumor regions of interest (ROIs) were manually selected based on the gadolinium contrast enhanced T1 weighted images (CET1W). A correlation matrix was generated to compare voxel-wise Spearman correlations between parameters within these ROIs.

## Results

**Figure 1:**
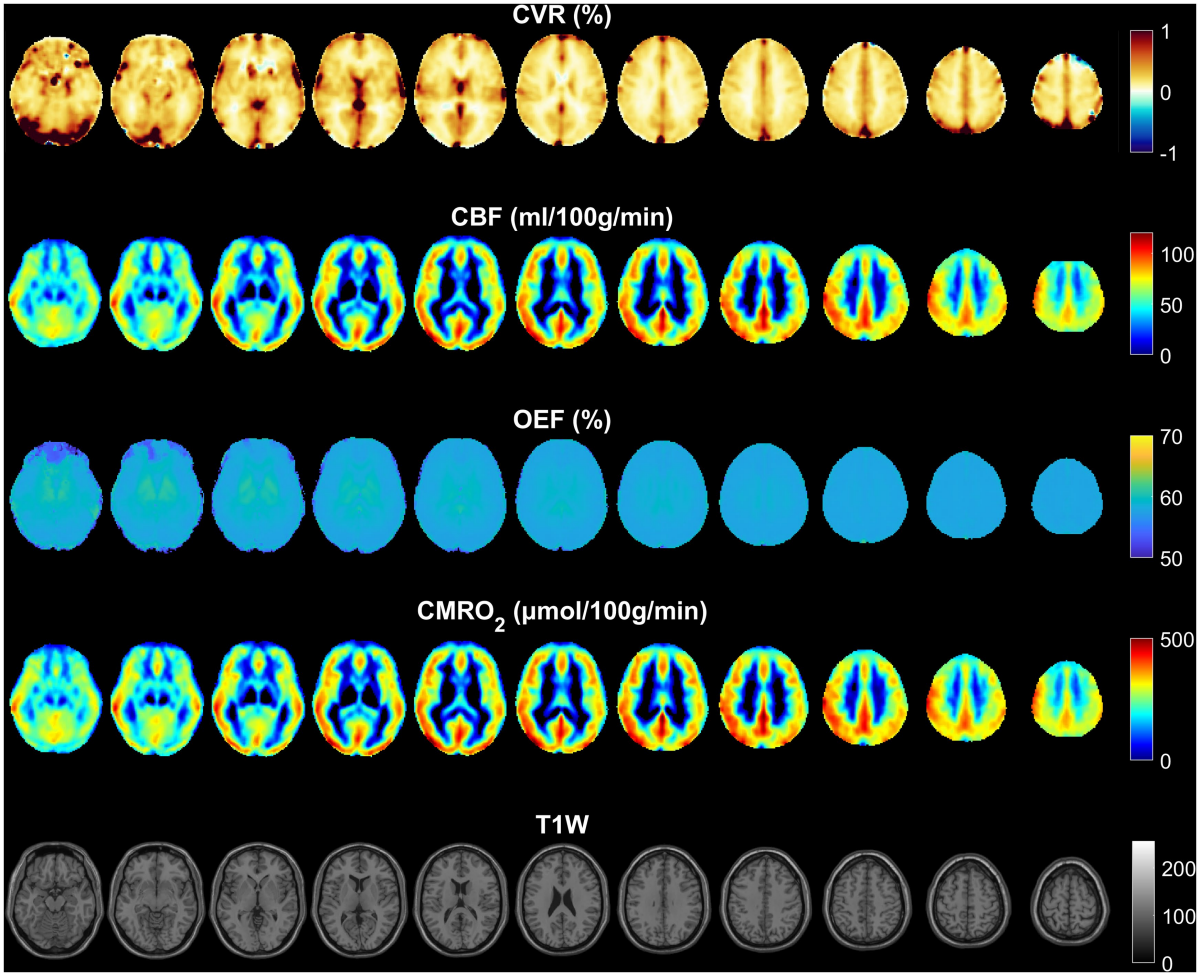
Normalized and averaged maps of CVR, CBF, OEF, and CMRO_2._ The distribution of CBF is similar to CMRO_2_, since the OEF is very homogeneous. The distribution of CVR is different from CBF and CMRO_2,_ especially in large draining veins.

Normalized and averaged maps of CVR, CBF, OEF and CMRO_2_ are presented in Figure 1. In the CVR maps, an increased value can be noticed at the location of large draining veins such as the superior sagittal sinus and the transverse sinus. The CBF and CMRO_2_ maps demonstrate a similar contrast, although with a different value range. OEF was homogeneous across most of the brain, but not at the center of the brain such as within the thalamus and putamen regions.

**Figure 2:**
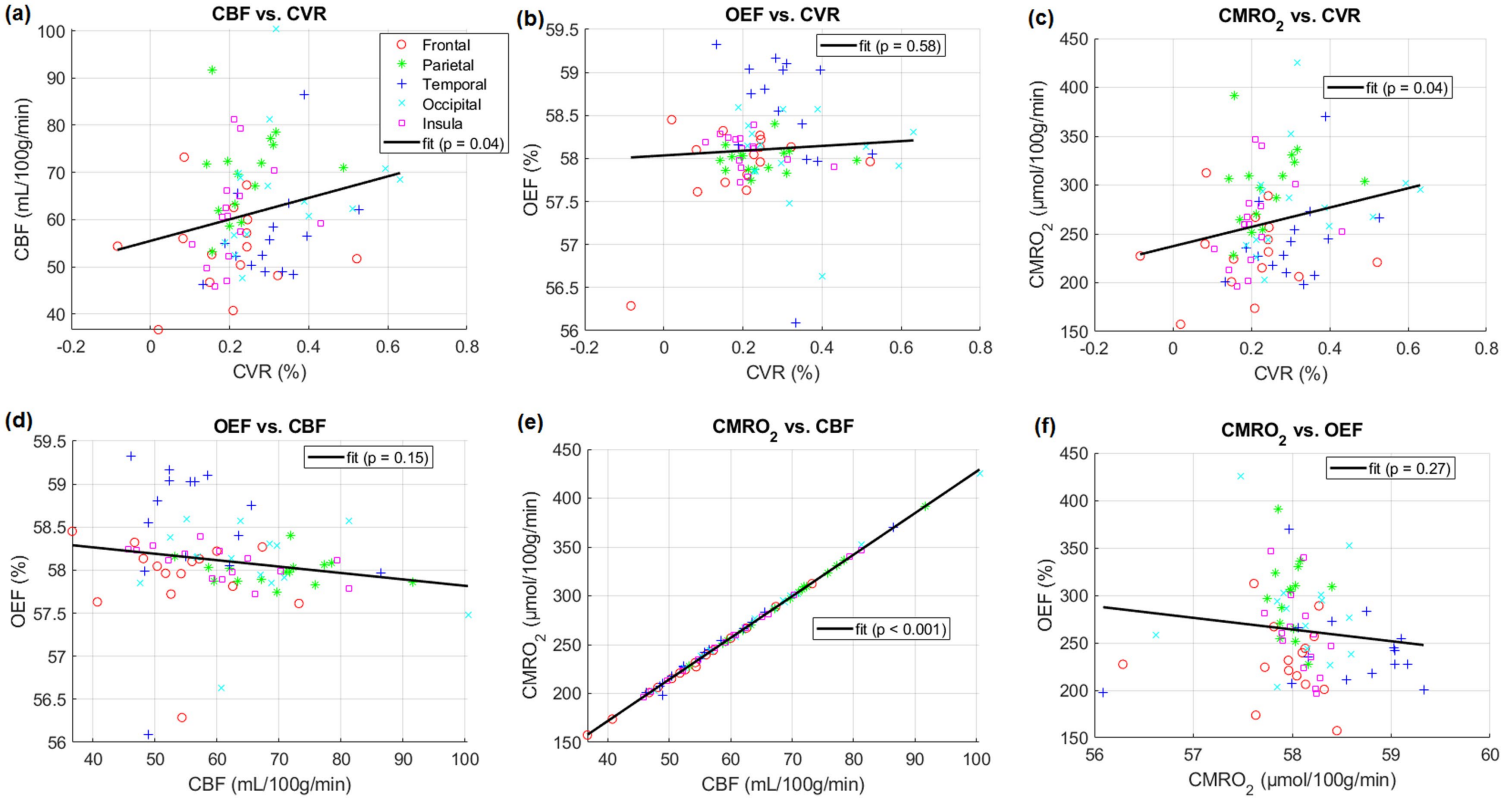
Comparisons between different physiological parameters averaged in listed five lobes of gray matter including frontal, parietal, temporal, occipital and insula lobes of individual subjects, respectively. Linear regressions and their parameters are included.

After linear regression, a positive correlation between CBF in five lobes and CVR across subjects was found (*p* < 0.05). Positive correlations between CMRO_2_ in the same regions and CVR across subjects were also found (*p* < 0.05). CBF and CMRO_2_ were almost perfectly correlated (*p* < 0.001, *r*^2^ = 1.0). However, correlations between OEF and other parameters were not significant. (*p* = 0.58 for OEF-CVR, *p* = 0.15 for OEF-CBF, *p* = 0.27 for OEF-CMRO_2_). Specific results are presented in Figure 2.

**Figure 3:**
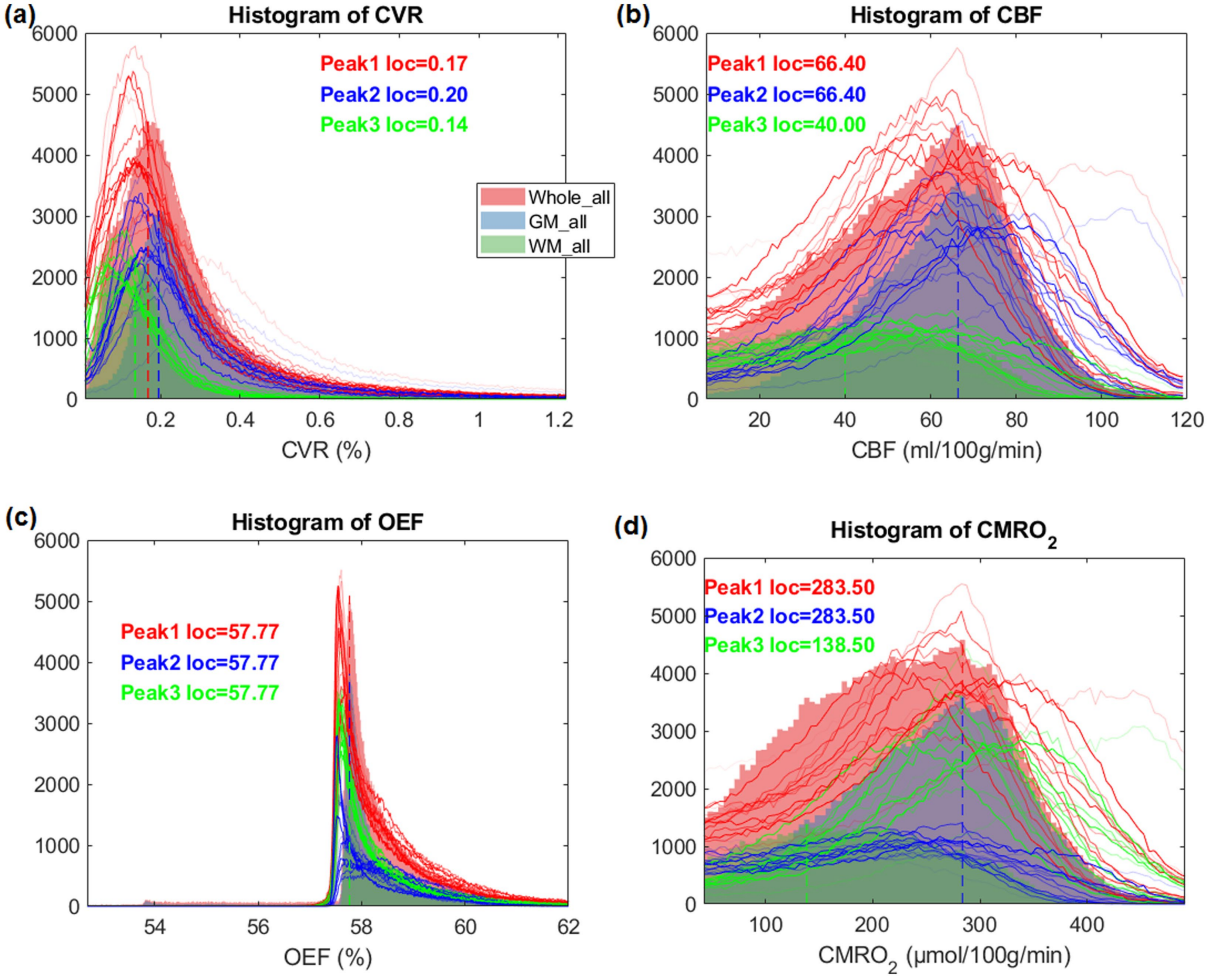
Histograms of CVR, CBF, OEF and CMRO_2_ maps, averaged for all heathy participants and individual subject (subject 1–15 as thin lines), in their whole brain, gray matter and white matter, respectively.

The histogram of CVR exhibited two peaks (GM and WM) in the intensity distribution (Figure 3). CBF and CMRO_2_ histograms were similarly distributed but showed a wider distribution. However, the histograms of OEF were very different and had a strong peak at the same location (≈57.8) both in the whole brain as well as within each of GM and WM separately.

**Figure 4:**
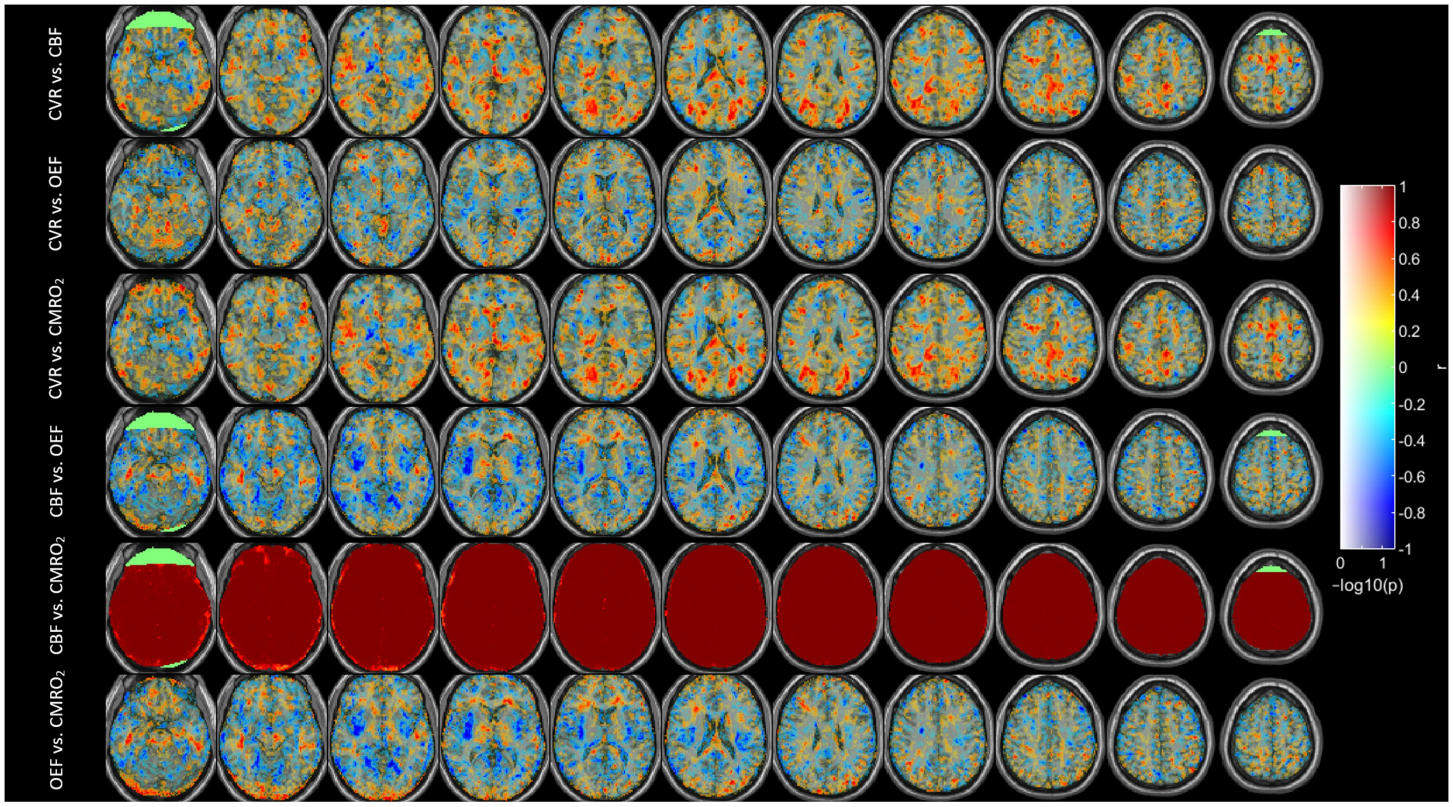
Correlation coefficients (r) between different parameters across subjects after using Spearman correlation. Correlation coefficients r is mapped to color as shown; −log10(p) is mapped to transparency. The range of -log10(p) was set to 0–1.3, corresponding to a *p*-value threshold of *p* < 0.05.

Figure 4 shows the Spearman correlation coefficients r between each pair among CVR, CBF, OEF and CMRO_2_. We saw strong positive correlations for CVR vs. OEF and CMRO_2_ in the white matter areas, especially for CVR vs. CMRO_2_ in the occipital region. On the other hand, some areas showed a strong negative correlation, e.g., CVR vs. OEF in the fronto-insular region or CBF vs. OEF in the insular region. A strong correlation between CBF and CMRO_2_ was found in the whole brain except for parts of the frontal region.

**Figure 5:**
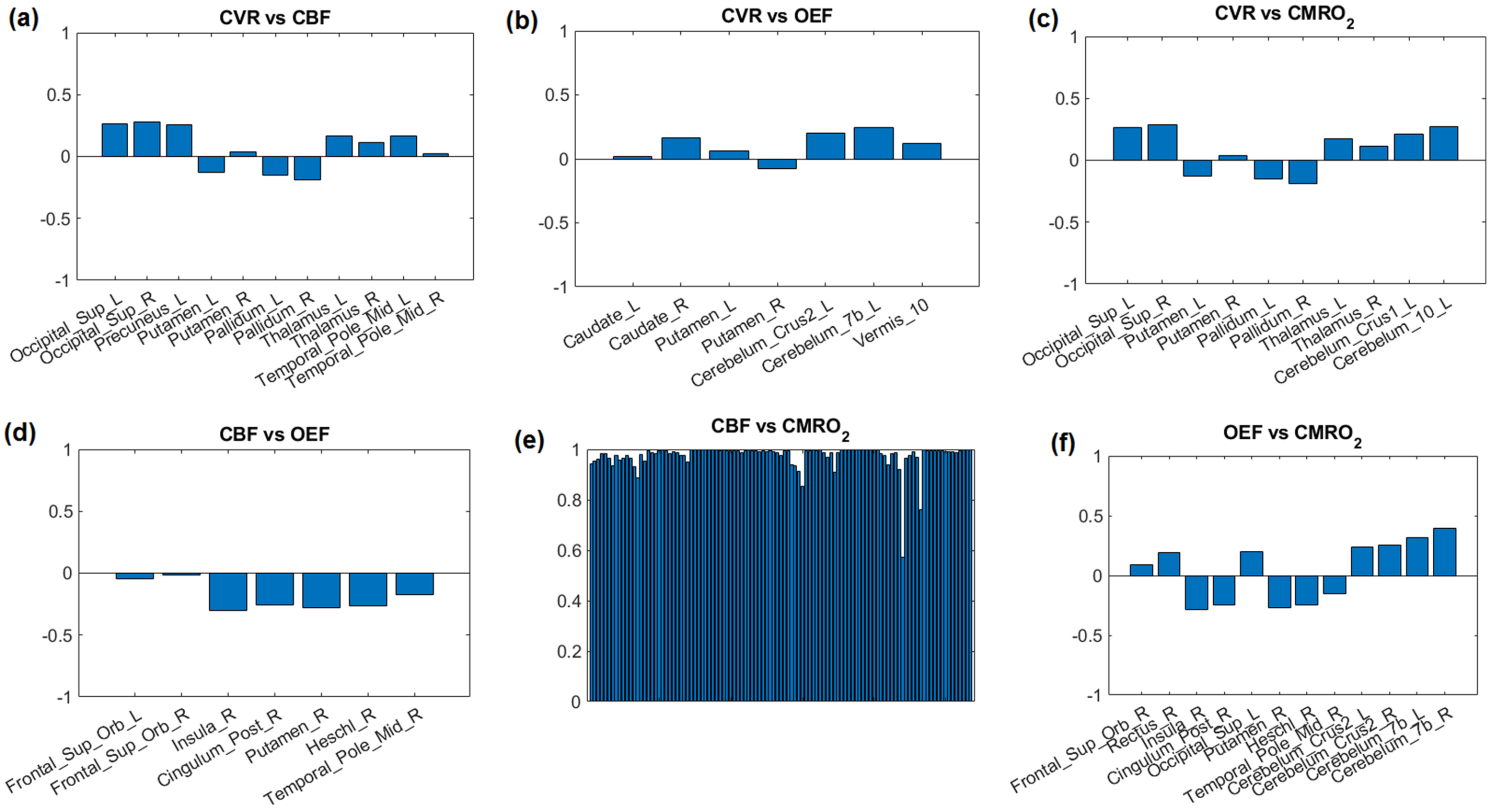
Correlation coefficients between parameter pairs are shown for the top 12 regions with the lowest average *p*-values. Due to the high correlation between CBF and CMRO_2,_ all 116 AAL regions were selected (E).

The correlations r of top 12 AAL regions with lowest *p*-value are shown in Figure 5. Due to the high correlation between CBF and CMRO_2,_ all 116 AAL regions were selected (Figure 5E).

**Figure 6:**
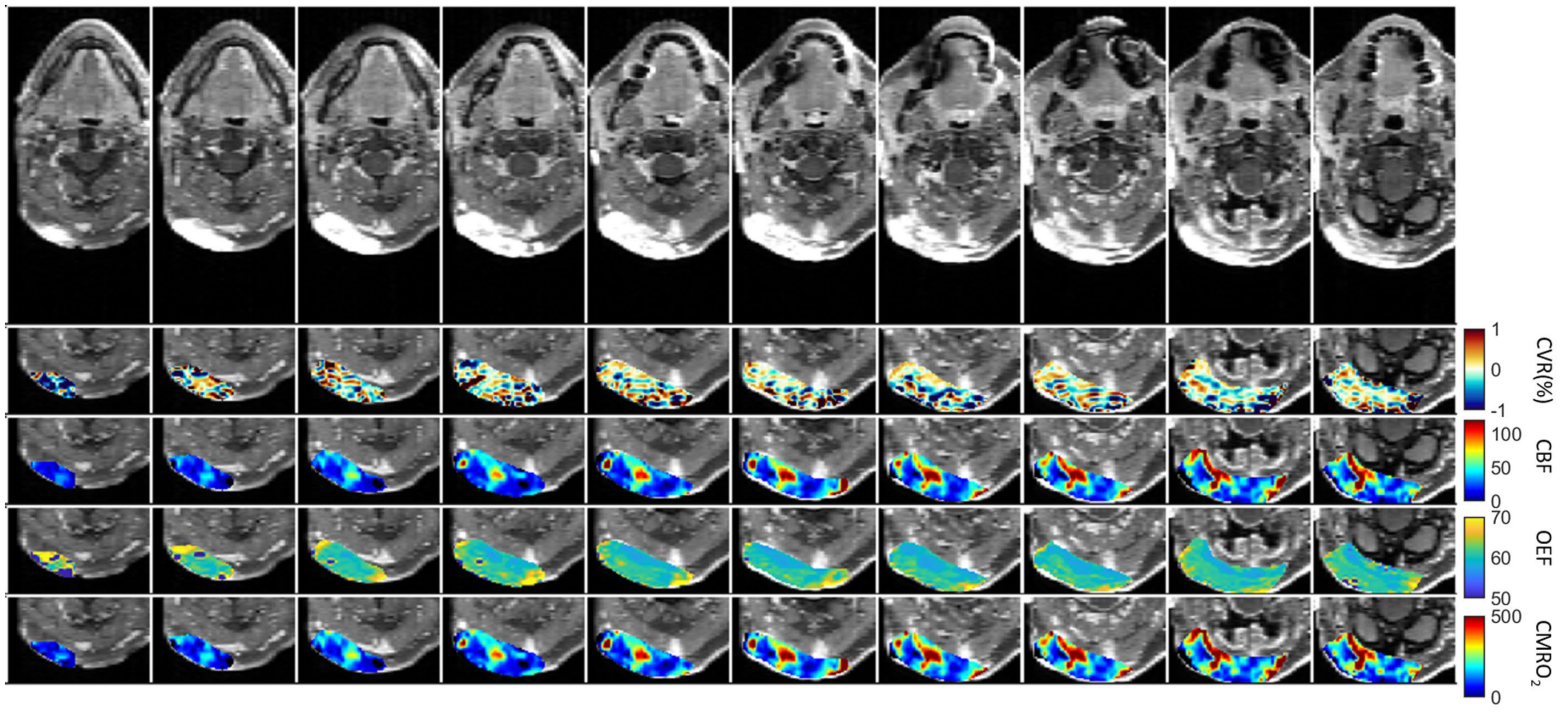
First patient with Melanoma (male, 59 years old, after therapy with Encorafenib und Binimetinib). The primary tumor was located at the right nuchal. ROI with abnormal CET1W were selected and different parameter maps within this ROI were overlaid.

The first patient with a brain tumor (melanoma) is presented in Figure 6. The primary tumor was located at the right nuchal. ROI with abnormal CET1W were selected and different parameter maps within this ROI were overlaid. The regions with high CBF showed lower OEF.

**Figure 7:**
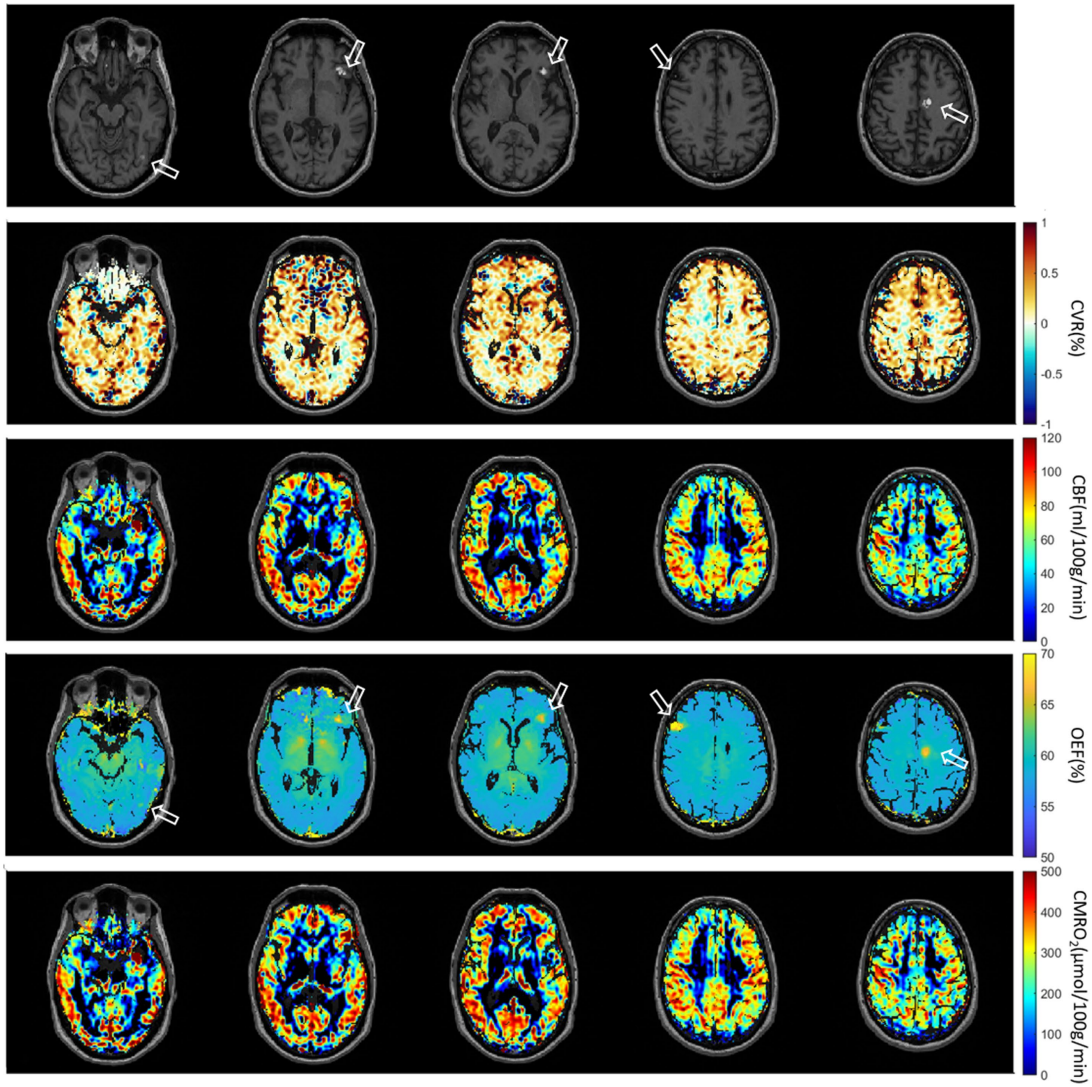
Second patient with Melanoma (male, 55 years old, after therapy with Nivolumab). The metastases are located at gyrus rectus on the left side (first column), in the frontal operculum on the left side (second column and third column), frontal lobe on the right side (fourth column) without CET1W-hyperintersities, and cingulate gyrus on the left side (fifth column).

The second patient with multiple brain metastases (melanoma) is presented in Figure 7. The metastases were located at gyrus rectus on the left side (first column), in the frontal operculum on the left side (second column and third column), frontal lobe on the right side (fourth column) without CET1W-hyperintersities, and cingulate gyrus on the left side (fifth column). Brain ROIs were masked and different parameter maps within the brain were overlaid on CET1W. The brain ROIs were defined according to CET1W. White matter was not intentionally excluded. Each parameter displayed abnormalities in different locations. OEF showed enhancements at the location with abnormal CET1W. CBF was increased at the first location but decreased at the last location. CVR was decreased in some metastasis locations.

**Figure 8:**
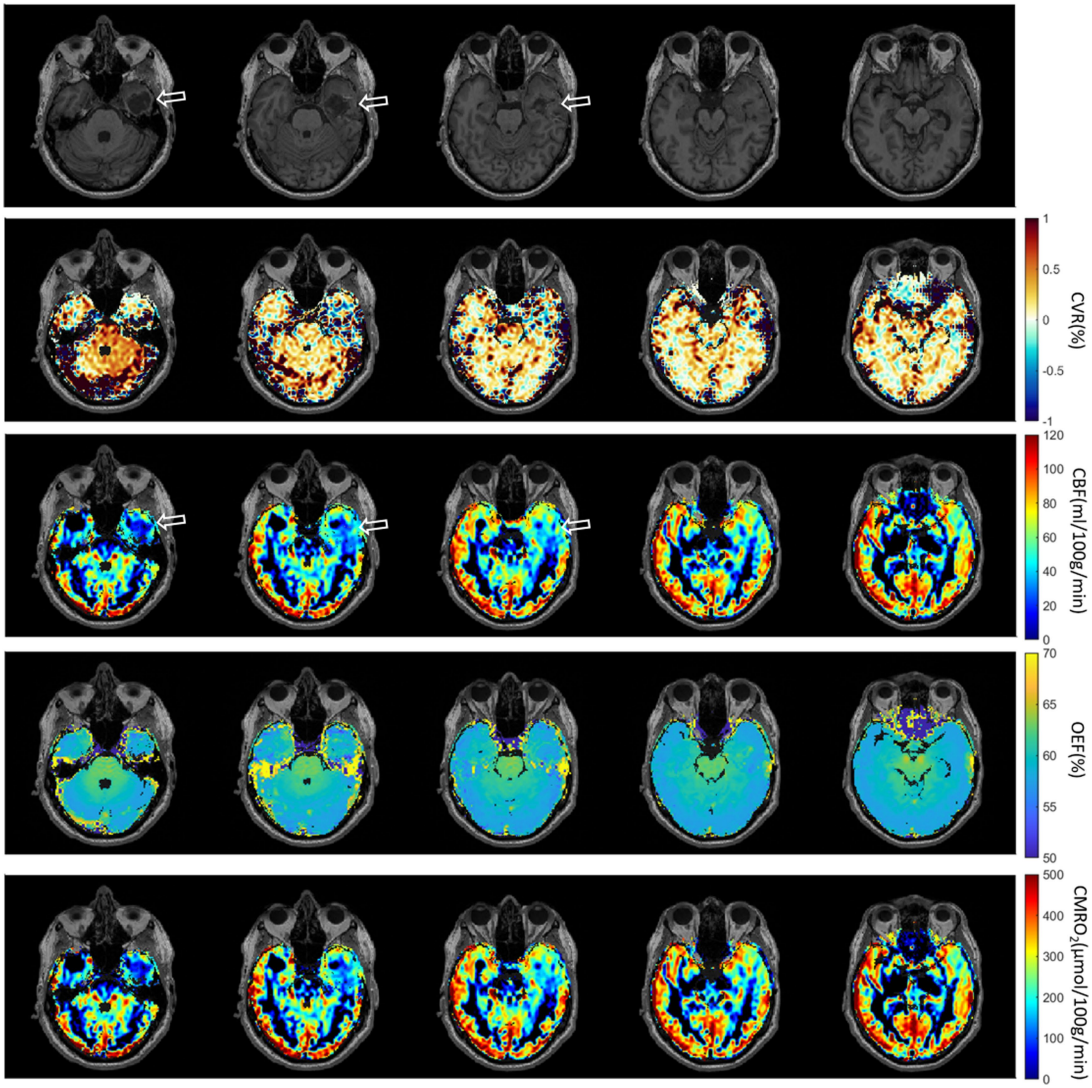
Third patient with brain Glioblastoma (male, 67 years old, after resection). The defect area after resection of a Glioblastoma in the left temporal lobe with infiltration of the adjacent dura could be observed.

The third patient with brain Glioblastoma is presented in Figure 8. A defect area after resection of a Glioblastoma in the left temporal lobe with infiltration of the adjacent dura could be observed. Brain ROIs were masked and different parameter maps within the brain were overlaid on CET1W. Generally, CBF and CMRO_2_ were decreased at the tumor location. CVR was increased at the lower tumor ring, as indicated in the first column.

Voxel-wise Spearman’s correlation coefficients between all pairs of physiological parameters in patient tumor ROIs are presented in Supplementary Figure S1. Except the strong correlation between CBF and CMRO_2,_ there was no correlation between other physiological pairs. The results of the remaining patients are presented in Supplementary Figures S2 and S3. Subject-specific maps of T1W, CVR, CBF and OEF from the first six subjects are presented in Supplementary Figure S4.

## Discussion

This study aimed to investigate how CVR derived from a breathing task relates to baseline CBF, OEF, and CMRO2. Consistent with previous reports, we found a significant positive correlation between CBF and CVR in five lobes of gray matter (Figure 2A) (6, 15, 17). Additionally, we found no significant correlation between OEF and CBF. This finding contradicts those of earlier research here OEF and CBF was found to be significantly correlated (*p* = 0.01) (7).

In this study, correlations between OEF and CVR in five lobes were not significant (Figure 2B). Significant positive correlations between CMRO_2_ and CVR in these lobes across subjects were also found (Figure 2C).

The CVR maps displayed increased values near the large draining veins, like the superior sagittal and transverse sinuses (Figure 1). These increased values were most likely a result of the interaction between sensitivity of the BOLD signal to blood oxygenation, proximity to large vessels, as well as partial volume effects, and the hemodynamic response in these regions. These elevated values do not necessarily reflect brain tissue reactivity but rather the influence of nearby venous structures. Increased OEF in central brain regions like the thalamus and putamen (Figure 1) may be driven by their high metabolic activity, specialized functions, and unique vascular supply characteristics. These areas have high oxygen demand due to their role in vital processes such as motor control, sensory relay, and synaptic transmission (26). Additionally, the vascular characteristics of these regions—small penetrating arteries and relatively lower blood flow—necessitate higher oxygen extraction to maintain efficient brain function (27, 28).

The lower or absence of correlation between OEF and other physiological parameters may arise from the fact that OEF primarily reflects the brain’s metabolic demand for oxygen, while other parameters like CBF and CVR are influenced by vascular and hemodynamic factors that do not always correspond directly to metabolic needs. Cerebral autoregulation, regional metabolic variation, and pathological states further contribute to the decoupling of OEF from these other physiological metrics.

The lack of correlation between CBF and OEF in this study, compared to previous studies (13, 14) could arise from measurement technique. Previous studies measured the global OEF in the superior sagittal sinus either using TRUST (14) or susceptometry-based oximetry (13). In global measurement, regional variations in these parameters could be averaged out, obscuring a relationship that might otherwise be seen in higher-resolution measurements. However according to Figures 1, 4, 5, different brain regions may exhibit varying relationships between CBF and OEF due to differences in metabolic demand. Areas with high baseline metabolism (e.g., the gray matter) could maintain a stable OEF despite fluctuations in CBF, while regions with lower metabolic demand would show less tight coupling. OEF maps (Figure 1) show relatively homogeneous distributions across much of the cortex, particularly within gray matter, consistent with stable oxygen extraction despite variations in perfusion. However, in subcortical regions such as the thalamus and putamen, areas known for high metabolic activity, OEF values appear elevated, likely due to increased oxygen demand. In contrast, CBF maps exhibit more spatial variability between gray and white matter regions, and between cortical areas. When these maps are considered alongside the CMRO₂ maps (which are derived from CBF × OEF), it becomes evident that CMRO_2_ variability is largely driven by CBF fluctuations, since OEF is relatively stable in the cortex. While strong correlations are observed between CBF and CMRO_2_ across most brain regions (Figure 4), correlations between OEF and either CBF or CMRO_2_ are weaker or absent, indicating that OEF remains relatively invariant in many areas despite variations in CBF. Figure 5 shows that very few regions met even a liberal p-threshold for significant correlation between OEF and CBF, reinforcing the observation that these parameters are uncoupled across much of the brain.

Except CBF and CMRO_2_, averaged maps (Figure 1) and corresponding intensity histograms (Figure 3) of physiological parameters revealed distinct distributions. Comparing to a previous study (29), the peak location of CVR in this study (0.17) is very similar. The histograms of CBF and CMRO2 were similarly distributed but showed a broader range. The peak location of CBF is around 65 mL/100 g/min which is close to the result of a previous study (30). However, the histograms of OEF were very different and had a strong peak at the same location in the whole brain as well as within each of GM and WM separately. Accordingly, we subsequently investigated the regional correlations between them.

CBF and CMRO_2_ are significantly correlated (Figure 4). This phenomenon is easily understandable since OEF is very homogenous and CMRO2 is the product of CBF and OEF. Therefore, CBF and CMRO_2_ are similarly distributed. The regional correlations between CVR, OEF, CBF, and CMRO₂ highlight the regional variability in how the brain regulates its blood supply and oxygen metabolism. Strong positive correlations in the white matter and occipital region suggest efficient matching of blood flow and oxygen metabolism, while negative correlations in areas like the insular region reveal compensatory mechanisms where blood flow and oxygen extraction are inversely related. In fMRI studies of visual stimulation (31), occipital regions showed coordinated increases in CBF, BOLD, and CMRO_2_ response. An inverse correlation of white matter CBF with connectivity has also been shown previously (32).

For regional correlations we selected the 12 AAL regions with the most significant correlations according to the lowest averaged *p*-value in each region (Figure 5). These regions could also be noticed in Figure 4.

Comparing to TRUST (8) we could measure full brain covered OEF with only minimal artifacts. The GESSE pulse sequence is a sequence that allows a hybrid mapping of T_2_ and T_2_^*^ relaxation times. It was developed to separate macroscopic and microscopic magnetic field inhomogeneities in MRI in microstructural brain imaging (18). In GESSE, a set of gradient echoes (GREs) are embedded around the spin echo (SE) of a single SE sequence (24). This sequence structure allows a simultaneous acquisition of a set of images corresponding to different GRE times (TEs) and therefore allows simultaneous T_2_ and T_2_^*^ mapping. The measurement of T_2_ and T_2_^*^ using GESSE was developed and applied for OEF mapping (23–25). GESSE is insensitive to RF pulse errors and does not suffer from significant field distortions, therefore providing a robust mapping technique in cerebral imaging. The range of OEF measured by qBOLD MRI typically lies between 0.2 and 0.5 (or 20–50%) under healthy physiological conditions (24). According to the histogram of OEF (Figure 3) the peak location is about 57.77 which is very close to the one in the literature (24).

In pathological conditions, the ability of blood vessels to widen in response to a stimulus may be compromised due to an already widened baseline, as seen in sickle cell disease where cerebral blood flow (CBF) is elevated and cerebrovascular reactivity (CVR) diminished (33, 34). In diseases characterized by narrowing or blockage of vessels, such as steno-occlusive diseases, a vasodilatory stimulus can increase CBF in regions with robust vasodilatory capacity. However, this may unexpectedly decrease CBF in neighboring areas with preserved or limited vasodilatory capacity (35). This phenomenon, known as “vascular steal,” may result in apparent negative CVR responses, either with or without changes in CBF (35). The relationship between CBF and CVR in pathological cases is often complex and not easily discernible. Similar to this principle, physiological parameters are distributed differently in the brain tumor cases (Figures 6–8). Except the strong correlation between CBF and CMRO_2,_ there was no correlation between other physiological parameters (Supplementary Figure S1). Due to the differences between physiological parameters, complete measurements of all of them will help the physician to discover different aspect of tumor properties. These metrics help physicians understand critical tumor characteristics such as vascularity, metabolic demand, hypoxia, aggressiveness, and response to treatment. By integrating these data, clinicians can develop more personalized and effective treatment plans, optimize therapeutic strategies, and improve prognosis assessment for patients with brain tumors.

We found that, except for CBF and CMRO2, no other parameters were correlated in tumor cases. This indicated that, apart from the fact that the small cohort of tumor patients may not permit a rigorous statistical analysis and interpretation, (i) local tumor blood flow is correlated with metabolic demand for both tumor and healthy brain regions, as already demonstrated in previous works (36, 37), and (ii) that cerebrovascular reactivity appears independent from other physiological parameters in the tumor region, suggesting a perturbed vascular architecture (38).

There are several limitations of this study. First, GESSE is a very long measurement that takes about 10 min. The versatility of combined GE and SE methodology has been demonstrated in various approaches, based on a wide range of sequence implementations, and differing in the readout (single- or multiple-line acquisitions), spatial resolution, number and type of echoes used (39). EPI outperforms single-line acquisitions in terms of acquisition time (TA) and temporal resolution, making it the current method of choice for fast imaging sequences. However, EPI suffers from distortion, blurring, and local signal loss (40). Secondly, CVR is measured with a breath-holding task. This task requires notable cooperation of patients or volunteers. Without gas challenges, CVR can also be measured during resting-state (41–43). However, special care needs to be taken for it to be more reliable (3, 16). The efficiency of breath-holding as a method for inducing hypercapnia needs to be correlated with physiological measurements like SPO₂ and blood CO_2_ levels to ensure adequacy. Without monitoring these, the induced hypercapnia may be inconsistent or inadequate, leading to potential bias in the measurement of CVR and other related parameters. This could undermine the reliability of the test results, highlighting the need for better understanding and standardization in CVR testing. While breath-holding is commonly used to assess CVR, its associated hemodynamic changes (like reduced venous return, altered cardiac output, and blood pressure fluctuations) can influence the results. These changes are sometimes monitored, but often they are not fully accounted for, which may introduce bias into the assessment of CVR parameters. To improve accuracy, more careful monitoring and control of these hemodynamic factors such as lagged-GLM^17^ for optimization are needed during CVR testing (44) and calculation. Thirdly, the ASL scan was a single PLD 3D pCASL. This sequence was not optimized for voxel-wise CBF measures in white matter (45). Finally, by using a constant *[H]_a_* in the CMRO_2_ equation, both the natural variance of and differences in hematocrit between biological sexes is neglected. This may significantly change the oxygenated heme molar concentration in arterioles. Additionally, the chemotherapy treatment in a participant with a metastatic brain could also significantly alter hematocrit.

## Conclusion

This study demonstrates a significant positive correlation between CVR and baseline CBF in five lobes of gray matter, indicating a close relationship between vascular reactivity and resting perfusion. In contrast, the weak or absent correlations between OEF and other parameters suggest that OEF reflects distinct metabolic processes less directly coupled to vascular dynamics. The observed regional variability in the relationships among CVR, CBF, OEF, and CMRO₂ highlights the heterogeneous nature of cerebral hemodynamics and oxygen metabolism.

## Data Availability

The data analyzed in this study is subject to the following licenses/restrictions: The data that support the findings of this study are available from the corresponding author upon reasonable request. Requests to access these datasets should be directed to ke.zhang@uni-heidelberg.de.

## Supplementary material

SUPPLEMENTARY FIGURE S1 Voxel-wise Spearman’s correlation coefficients between all pairs of physiological parameters in the tumor ROIs from patient 1 **(a)**, patient 2 **(b)** and patient 3 **(c)**.

SUPPLEMENTARY FIGURE S2 Fourth patient with metastatic Melanoma (male, 67 years old, after radiotherapy).

SUPPLEMENTARY FIGURE S3 Fifth patient with metastatic Melanoma (male, 40 years old, after radiotherapy).

SUPPLEMENTARY FIGURE S4 Individual T1 weighted anatomical, CVR, CBF and OEF maps were calculated in all subjects (left-right).

SUPPLEMENTARY FIGURE S5 Breath-holding task design—five blocks, each consisting of 10 repetition of free breathing (FB), 10 repetition of breath-hold (BH), TR is equal to 1.7s.
