# Supplementary figures and images for: Correlation of breathing task derived cerebrovascular reactivity with baseline CBF, OEF and CMRO2
Source: Front Neurol. 2025 Oct 10;16:1534844. doi: 10.3389/fneur.2025.1534844 (PMC12549280; doi:10.3389/fneur.2025.1534844)

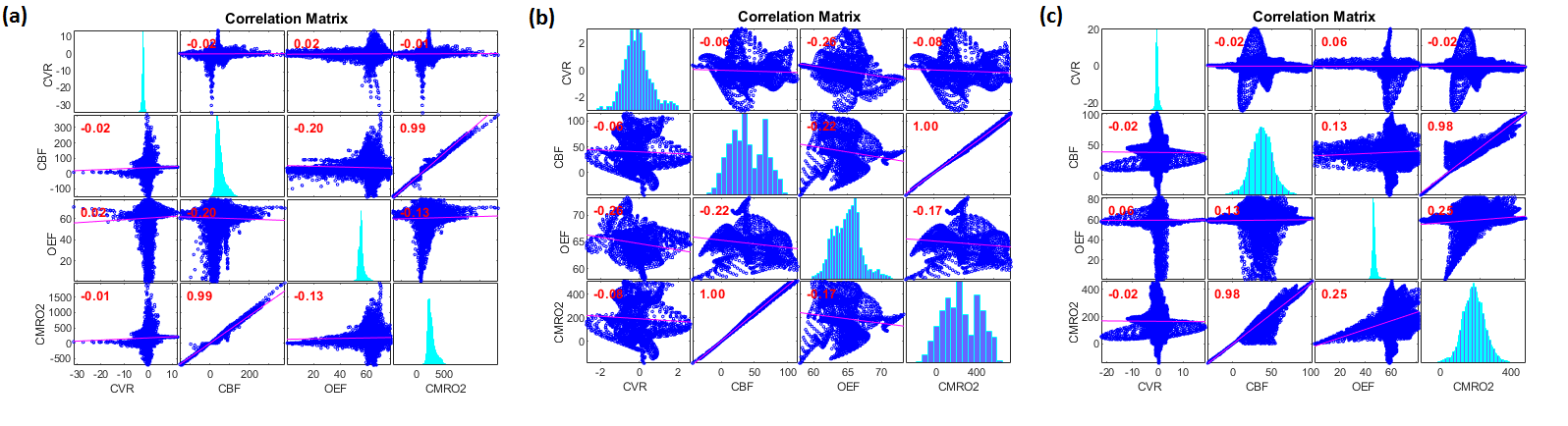

Supplement: SUPPLEMENTARY FIGURE S1 — Voxel-wise Spearman’s correlation coefficients between all pairs of physiological parameters in the tumor ROIs from patient 1 (a), patient 2 (b) and patient 3 (c). [file Image_1.TIF]

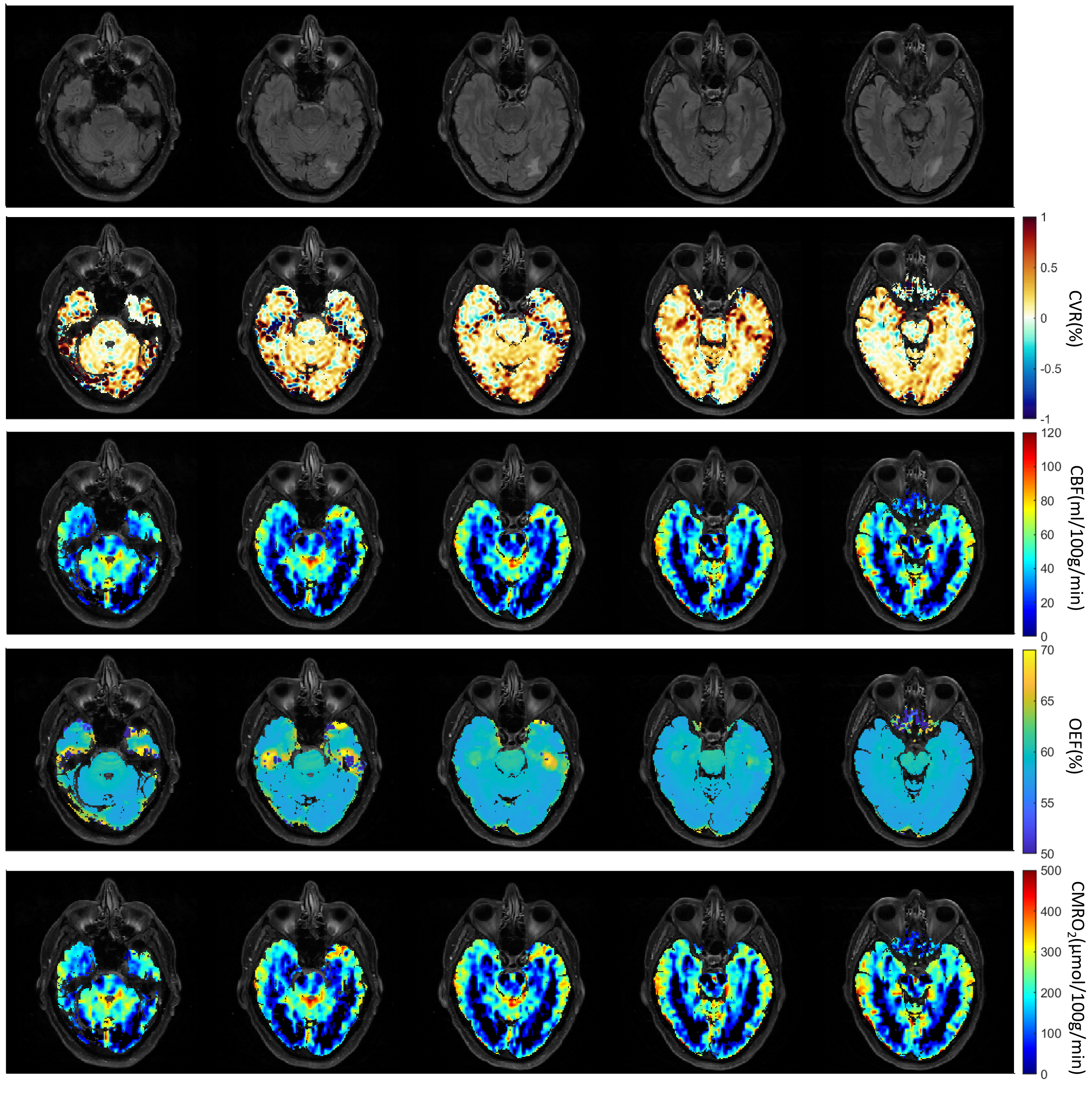

Supplement: SUPPLEMENTARY FIGURE S2 — Fourth patient with metastatic Melanoma (male, 67 years old, after radiotherapy). [file Image_2.TIF]

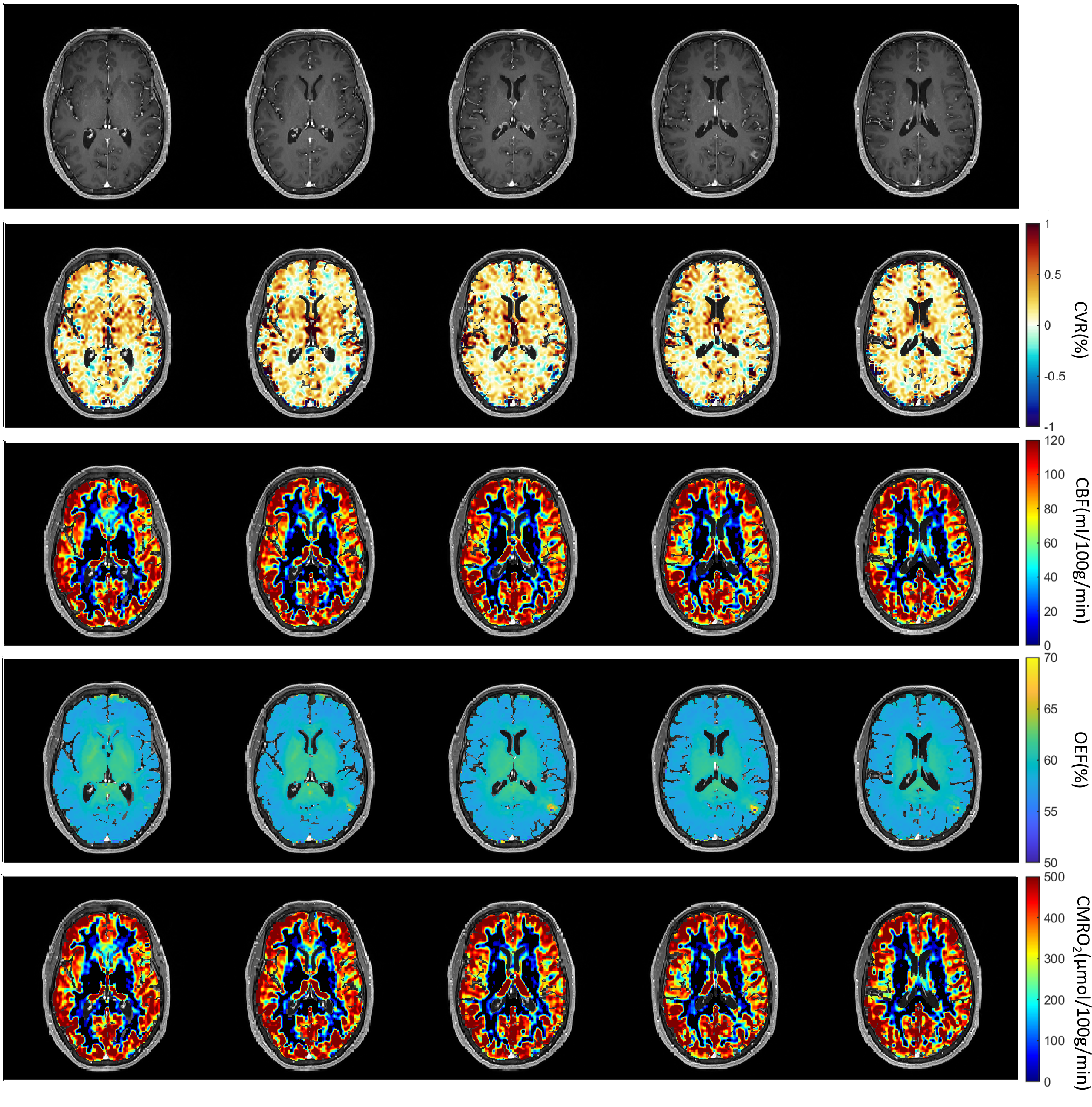

Supplement: SUPPLEMENTARY FIGURE S3 — Fifth patient with metastatic Melanoma (male, 40 years old, after radiotherapy). [file Image_3.TIF]

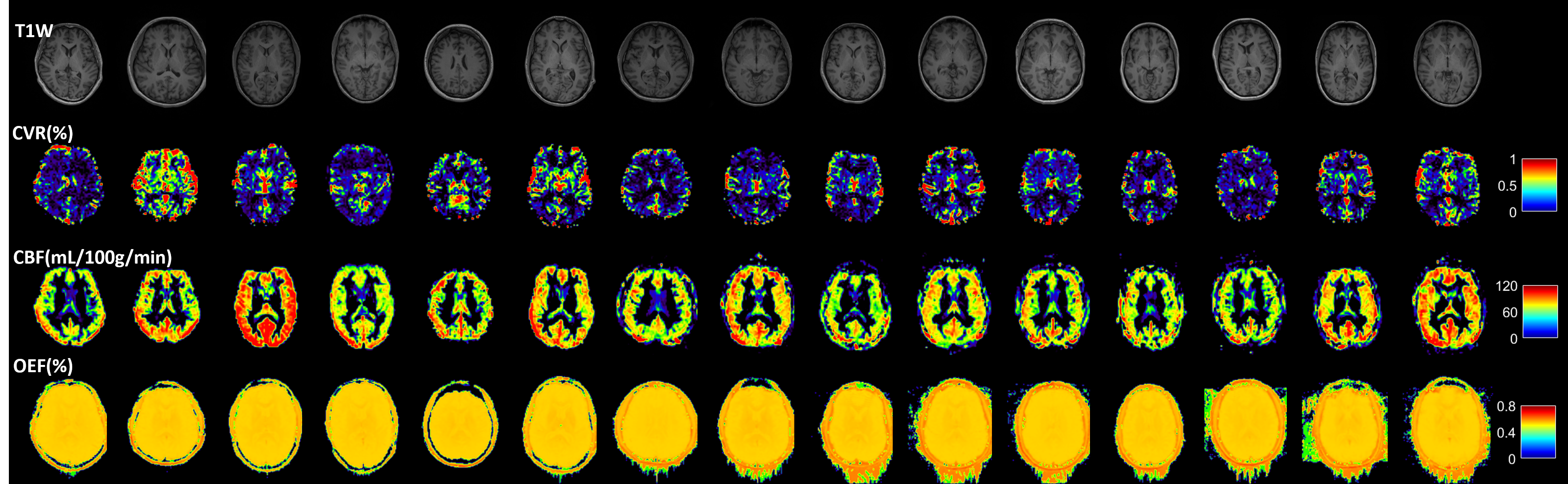

Supplement: SUPPLEMENTARY FIGURE S4 — Individual T1 weighted anatomical, CVR, CBF and OEF maps were calculated in all subjects (left-right). [file Image_4.TIF]

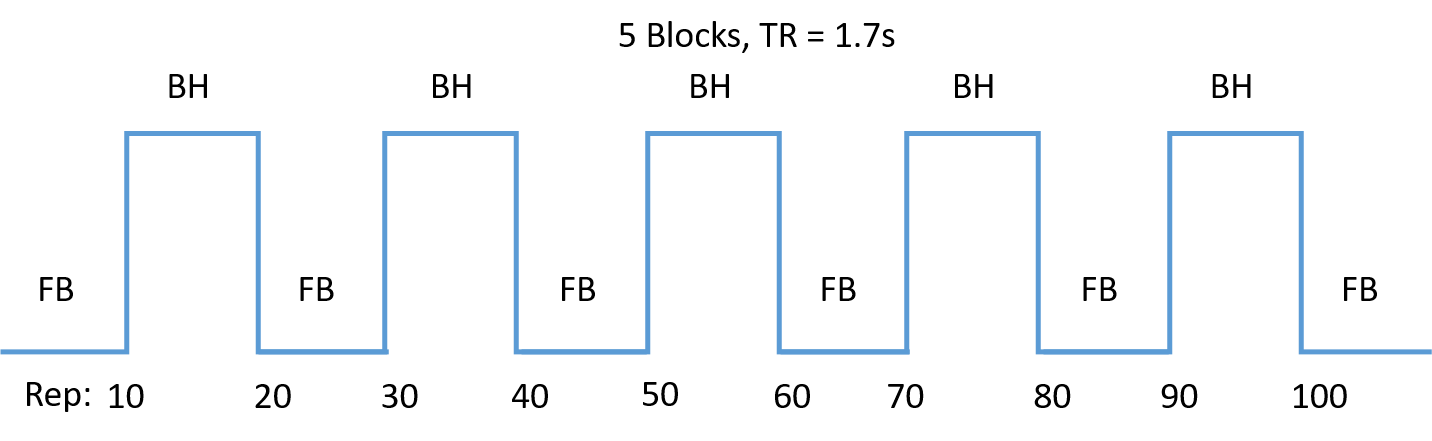

Supplement: SUPPLEMENTARY FIGURE S5 — Breath-holding task design—five blocks, each consisting of 10 repetition of free breathing (FB), 10 repetition of breath-hold (BH), TR is equal to 1.7s. [file Image_5.TIF]
